# Supplementary figures and images for: Definitions matter: Heterogeneity of COVID-19 disease severity criteria and incomplete reporting compromise meta-analysis
Source: PLOS Glob Public Health. 2022 Jul 19;2(7):e0000561. doi: 10.1371/journal.pgph.0000561 (PMC10021556; doi:10.1371/journal.pgph.0000561)

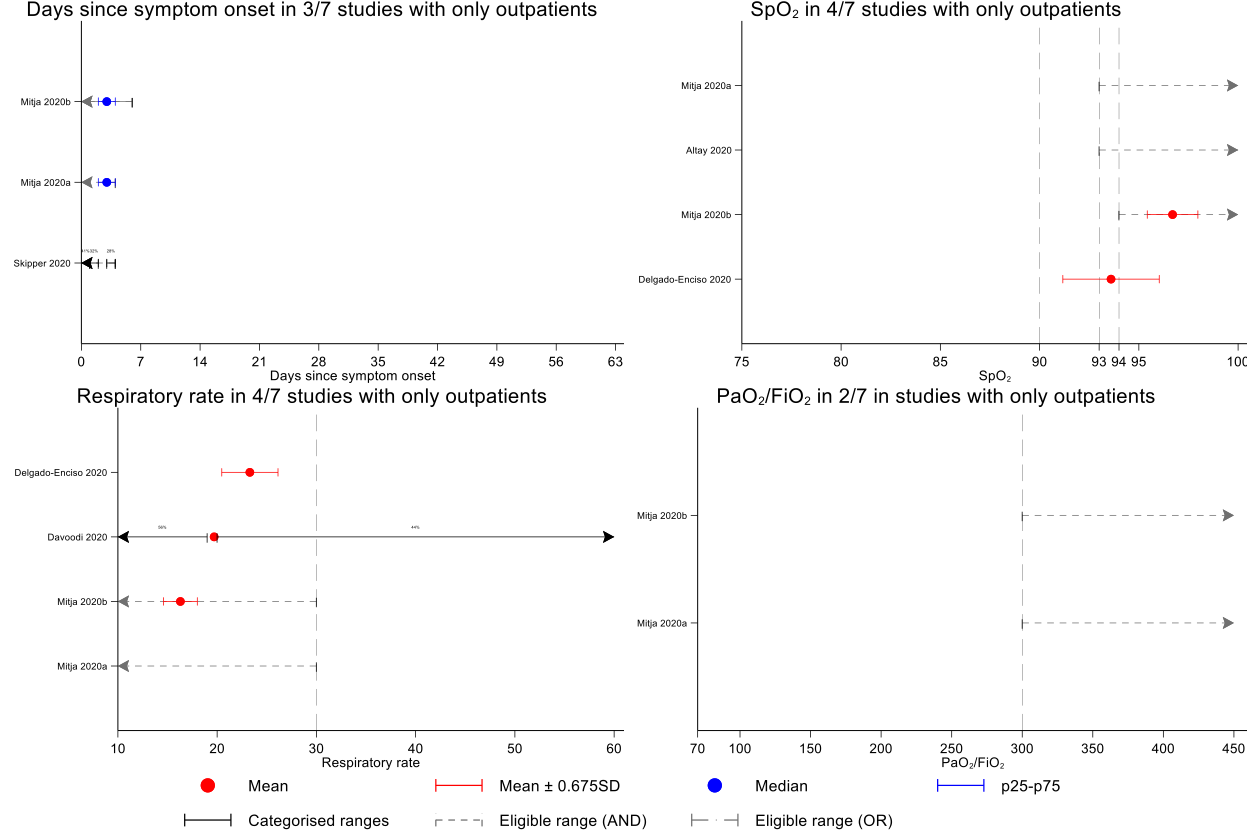

Supplement: S1 Fig — Dashed vertical lines denote thresholds used by either the NIH; WHO; Covid-NMA or Magic NMA groups to categorise severity. Eligible range (AND) refers to a criterion that is part of an AND condition and therefore an individual falling into this range would also require at least one other criterion to be met; eligible range (OR) refers to a criterion that is part of an OR condition. SpO2: Blood Oxygen Saturation; PaO2/FiO2: ratio of arterial oxygen partial pressure (PaO2 in mmHg) to fractional inspired oxygen (FiO2 expressed as a fraction)). (TIFF) [file pgph.0000561.s001.tiff]

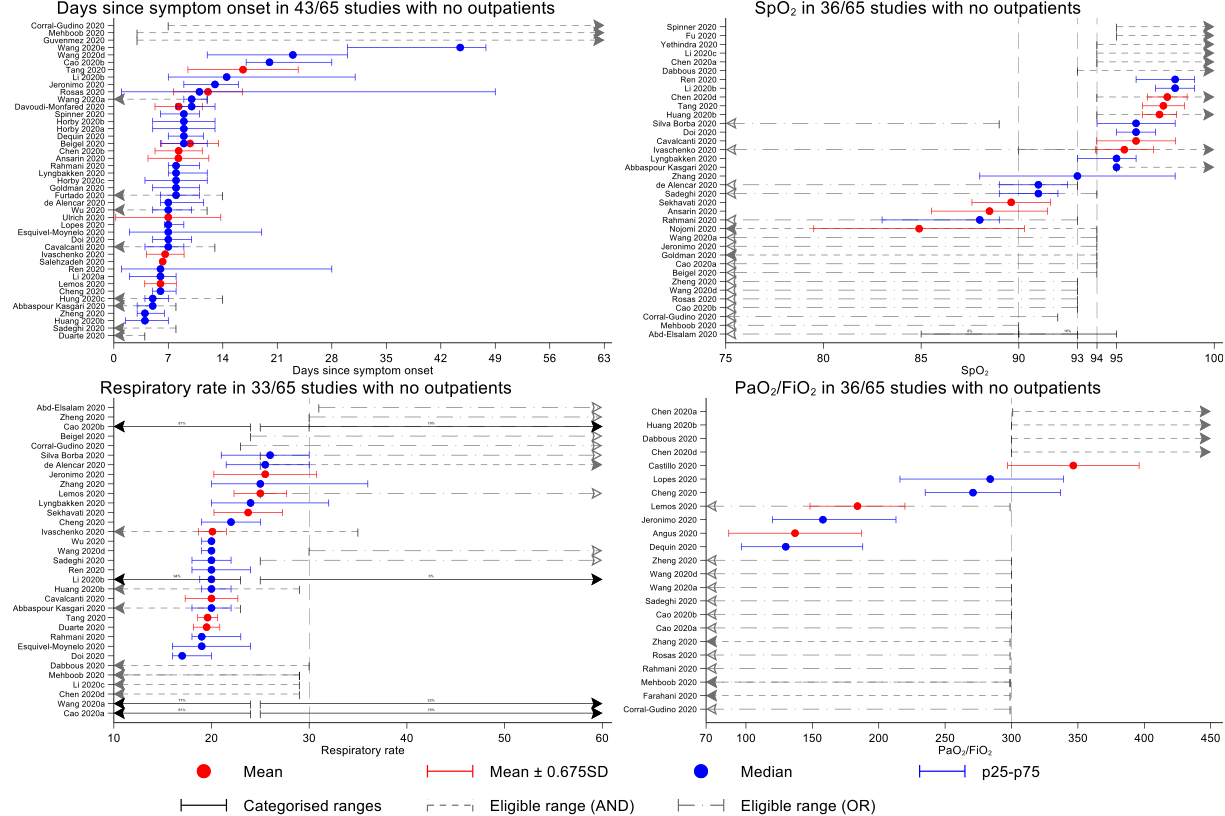

Supplement: S2 Fig — Dashed vertical lines denote thresholds used by either the NIH; WHO; Covid-NMA or Magic NMA groups to categorise severity. Eligible range (AND) refers to a criterion that is part of an AND condition and therefore an individual falling into this range would also require at least one other criterion to be met; eligible range (OR) refers to a criterion that is part of an OR condition. SpO2: Blood Oxygen Saturation; PaO2/FiO2: ratio of arterial oxygen partial pressure (PaO2 in mmHg) to fractional inspired oxygen (FiO2 expressed as a fraction)). (TIFF) [file pgph.0000561.s002.tiff]

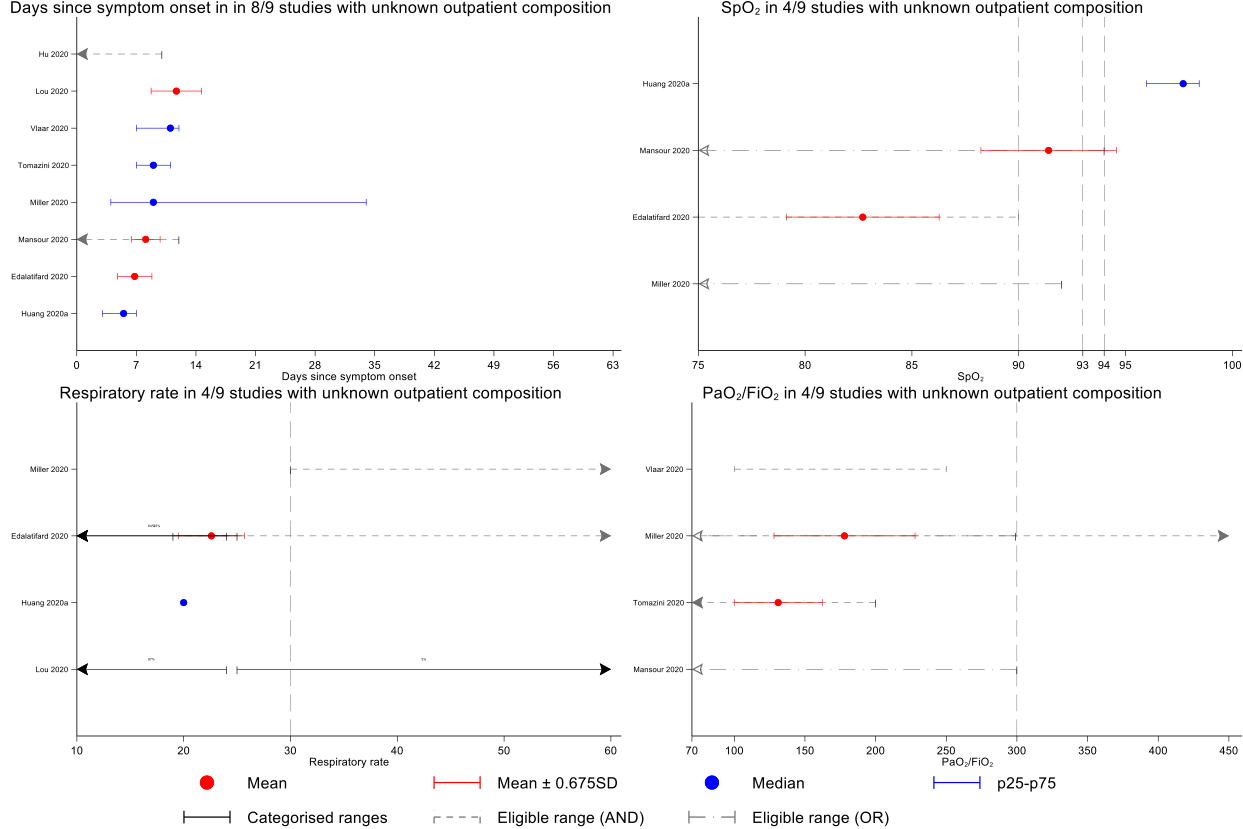

Supplement: S3 Fig — Dashed vertical lines denote thresholds used by either the NIH; WHO; Covid-NMA or Magic NMA groups to categorise severity. Eligible range (AND) refers to a criterion that is part of an AND condition and therefore an individual falling into this range would also require at least one other criterion to be met; eligible range (OR) refers to a criterion that is part of an OR condition. SpO2: Blood Oxygen Saturation; PaO2/FiO2: ratio of arterial oxygen partial pressure (PaO2 in mmHg) to fractional inspired oxygen (FiO2 expressed as a fraction)). (TIFF) [file pgph.0000561.s003.tiff]
