# Supplementary material for: Definitions matter: Heterogeneity of COVID-19 disease severity criteria and incomplete reporting compromise meta-analysis
Source: PLOS Glob Public Health. 2022 Jul 19;2(7):e0000561. doi: 10.1371/journal.pgph.0000561 (PMC10021556; doi:10.1371/journal.pgph.0000561)
Supplement: S3 Table — (DOCX) [file pgph.0000561.s006.docx]

**Supplementary Table 3.** Agreement between WHO - COVID-19 Living Network Meta-Analysis and COVID-NMA initiative groups with respect to possible minimum and maximum severity of trial participants (n=70)

|  | **Discordant maximum severity** | **Concordant maximum severity** |
| --- | --- | --- |
| **Discordant minimum severity** | 14 (20%) | 15 (21%) |
| **Concordant minimum severity** | 15 (21%) | 26 (37%) |
