# Supplementary material for: Definitions matter: Heterogeneity of COVID-19 disease severity criteria and incomplete reporting compromise meta-analysis
Source: PLOS Glob Public Health. 2022 Jul 19;2(7):e0000561. doi: 10.1371/journal.pgph.0000561 (PMC10021556; doi:10.1371/journal.pgph.0000561)
Supplement: Attachment — Submitted filename: Reponse to reviewers_230522_Plos Global Health_submitted.docx [file pgph.0000561.s007.docx]

**Response to reviewers**

Reviewer 1.
Overall: This is a timely and informative study. The authors carefully reviewed the current treatment guidelines and the studies used to inform the guidelines and provided logical and evidence-based critiques on the inconsistency in definitions/categorizations of COVID 19 disease severity. I (the reviewer) consider myself as a population health researcher with limited clinical experience, but I may be able to provide perspectives that are representative of the potential readers of the journal. Another overall comment is that the figures in this study are very informative, but I am afraid they are not self-explanatory enough. In general, more detailed notes/legends will be very helpful. Please consider the following recommendations.

(I found it very inconvenient to write comments without the availability of line numbers. Please consider adding line numbers if the manuscript needs reviews in the future)

*Response from the authors: We thank the reviewer for his/her valuable comments. The notes/legends of each figure were at the end of the manuscript and not next to the figures and might have been missed. We have added the line number in the revised version.*

Introduction:

(1) The concepts of “disease severity” and “stage of the disease” have been used interchangeably in the current study. But to me, the stage of the disease seems to be more related to time/phase instead of severity, especially when being used to determine, for example, whether a patient is at the initial infection phase of the disease where the viral burden peaks, or at the later phase when inflammatory processes dominate. Further clarifying these concepts is needed because the introduction section particularly emphasized the importance of determining disease course, and how crucial it is in terms of therapeutic recommendations (the timing of when to administer medicines). In the intro, the authors may consider at least briefly answering why a more consistent and more precise severity assessment can help better identify the stage of the disease, and thus guide and improve therapeutic recommendations.

*Response from the authors: We have removed the concept of stage of the disease, which can be equally reported as “disease severity”, and avoid introducing a similar concept. We believe that the sentences from lines 72 to 77 explain in medical terms the evolution of the disease and the rationale for using different therapy.*

(2) Though already specified in the main text, from a reader’s perspective, it is still helpful and necessary to have notes explaining the abbreviation used in the figures. (For example, Figure 1, respiratory rate and oxygen saturation level.)

*Response from the authors: Acronym explanations have been added in the legend of figure 1 and 5*

(3) In the last paragraph of the introduction, the aim of the study is phrased “We sought to determine the feasibility of determining retrospectively the disease severity of patients included in the COVID-19 clinical trials upon which the current WHO living therapeutic guidelines are based.” I personally think that this sentence can be potentially reframed and simplified. Also, the authors may reconsider whether the aim of the study is determining the feasibility of the current measures, or examining the consistency or comparability of the current measures.

*Response from the authors: We thank the reviewer for his/her comment. We respectfully argue that the study's goal is correctly described in the current sentence and prefer not to change it.*

Materials and Methods:

(4) Under the subtitle of “categorical indicators of disease severity”, since the categories indicate severity, it will be very helpful if they are ordered accordingly, starting from the least severe category to the most severe one.

*Response from the authors: We have reorganised the severity category from the least to more severe form*

Results:

(5) Figure 2 needs notes/legends. Please specify the meaning of red dots and green triangles. Please specify the difference between the red and black dash lines.

*Response from the authors: The meaning of red dots and the green triangle is already documented in the legend line 415-417*

(6) Figure 3 needs notes/legends and reorganization. Please specify the meaning of the colored dots. Please relocate the letter A and letter B, so they do not overlap with the figure title.

*Response from the authors: The meaning of coloured dots is already documented in the legend line 419-424. We will work with the editor to avoid the overlap of the letters and title.*

(7) Figure 5 needs notes/legends. Please specify what the different colored arrows mean.

*Response from the authors: The meaning of red dots and green triangles is already documented in the legend line 429-436 and in the graph itself.*

Discussion:

(8) The paper clearly pointed out the drawbacks of the current severity measurement. It might be obvious to the authors that how individual patient data meta-analysis will be able to contribute, but to people who are not very familiar with clinical research, more details on how the proposed solution will provide evidence for a consistent and accurate severity definition will be extremely helpful. In other words, I do think the current research revealed very important issues of the current literature, but I do not think it indicates the future direction of the research clearly enough.

*Response from the authors: We thank the reviewer for his/her comment. We have added an explanation about the value of* individual patient data meta-analyses, lines 312-329.

Reviewer 2
This is a cleverly conceived methods paper which highlights the difficulties of both setting up trials for a new disease in the absence of clear understanding of the disease pathogenesis, and how interventions might vary in effectiveness at different disease stages, and making recommendations on therapeutics at a global level. It is useful to highlight how the deficiencies described in the paper have compromised meta-analysis and recommendations, but I would like the authors to expand the discussion on why this has happened, and strategies so that it would not happen again in future pandemics.

The abstract recommendations are a logical conclusion from the findings, but could there be a final line on how this could be achieved?

*Response from the authors: We thank the reviewer for his/her comment. We have added recommendations in the abstract, line 41-44.*

I think it would be useful to have the individual studies that have been used to generate the meta-analytic findings are listed as references in a supplement. There are no descriptions of what software was used to generate the figures and calculate the summary statistics, so it would be useful to include this information in the methods.

*Response from the authors: The list of the 81 references is reported in supplementary table 2. A paragraph on statistic has been added lines 176-178.*

I agree with the authors that standard definitions is a very important initiative, but given that authors have not reported key clinical measures used in their studies, could the authors propose a strategy for achieving this? Poor reporting has been noted for many years, and the equator-network has sought to develop guidelines to improve reporting standards.

Many journals have accepted the manuscripts with incomplete reporting of key clinical measures, even though CONSORT and other reporting guidelines exist, and these guidelines ask for eligibility criteria, completely defined pre-specified primary and secondary outcome measures, including how and when they were assessed. Part of the problem is therefore manuscripts accepting reports, when the ideal information in the reports is not given. For journals that ask require reporting guideline checklists, this responsibility ultimately is a failure of the editorial and review process. The discussion should note these deficiencies, and suggest a strategy for improvement.

*Response from the authors: We agree with this statement but this review did not assess the compliance to reporting guidelines. We added a sentence in the discussion making the point raised by the reviewer. Line 290-294.*

I suspect some of this poor reporting reflects the haste of authors to publish their work, and the haste of journals to publish comparatively new information. International collaboration has been poor in many aspects of this pandemic, including investigating viral origins, and ensuring equitable access to vaccines.

I also agree that international collaborations are required to ensure equitable data sharing. Some, but not all journals require that data is available in a public registry, and authors are often reluctant to agree to this demand. Do the authors have suggestions on how this aspect could be improved?

*Response from the authors: We agree on the lack of adequate data sharing and enforcement of this policy. A chapter has been added to the discussion to illustrate this point. Line 312-329*
